# Mixed protein-templated luminescent metal clusters (Au and Pt) for H_2_O_2_ sensing

**DOI:** 10.1186/1556-276X-8-182

**Published:** 2013-04-19

**Authors:** Min Li, Da-Peng Yang, Xiansong Wang, Jianxin Lu, Daxiang Cui

**Affiliations:** 1 Key Laboratory of Laboratory Medicine, Ministry of Education, Zhejiang Provincial Key Laboratory of Medical Genetics, Wenzhou Medical College, Wenzhou, Zhejiang, 325035, China; 2 Department of Bio-Nano Science and Engineering, Key Laboratory for Thin Film and Microfabrication of Ministry of Education, Institute of Micro-Nano Science and Technology, Shanghai Jiao Tong University, 800 Dongchuan Road, Shanghai, 200240, People’s Republic of China

**Keywords:** Metal clusters, Mixed protein, H_2_O_2_ sensing, Biomineralization, Green synthesis

## Abstract

A simple and cost-effective method to synthesize the luminescent noble metal clusters (Au and Pt) in chicken egg white aqueous solution at room temperature is reported. The red-emitting Au cluster is used as fluorescent probe for sensitive detection of H_2_O_2_.

## Background

Owing to their ultra-small size, good biocompatibility and intriguing physicochemical properties, noble metal clusters show significant promise in biolabeling/bioimaging, sensing, catalysis, and optoelectronic nanodevices [1-7]. In general, there are two pathways to synthesize these fascinating materials: chemical and biological methods. The chemical method mainly includes (1) monolayer-protected method [8], (2) ligand etching method [9], (3) protection-deprotection method [10], and (4) template-assisted method [11]. Although atomically precise clusters with different species have been successfully obtained by these methods, from the ‘12 principles of developing green chemistry,’ there are still many problems to be resolved, such as the elaborate preparation procedure, the heavy use of organic solutes and/or surfactants and/or hazardous regents, and the high reaction temperature and long reaction times [12]. Compared with the chemical method, the biological method particularly refers to the template method, which is inspired by biomineralization behavior of organisms in nature. A variety of biomolecules such as amino acids [13], peptides [14], DNA [15,16], and proteins [17] have been used as template or scaffold to synthesize noble metal clusters. Among them, protein-protected luminescent noble metal clusters are of particular interest due to their simple preparation and potential applications [18]. Up to now, some proteins (including bovine serum albumin [19-23], lysozyme [24], transferrin family protein [18], human serum transferrin [25], pepsin [26]) have been widely explored to synthesize noble metal clusters. However, most proteins used are expensive, which hinders their further development in preparing production-level commercial-scale materials. It is worth noting that Shao et al. successfully synthesized Au and Ag clusters by using a kind of cheap materials - egg shell membrane - as template [27]. However, the use of hazardous reducer (NaBH_4_) and special treatment (UV illumination) is not environmentally friendly. In addition, the resulting products existing only in the form of a solid state greatly hinder their wide-range use. In order to satisfy the trends of developing green nanoscience and industrial production [12], a simple, green, cost-effective, and flexible strategy of preparing noble metal clusters is urgently required.

## Methods

Inspired by the work of Shao et al., herein, we report a one-pot green process to synthesize noble metal clusters (Au and Pt) with different luminescences by using chicken egg white as templates at room temperature in aqueous solution. Compared with the existing methods, the egg white-templated synthesis has several prominent advantages: (a) the raw material (chicken egg white) is easily available and rather cheap; (b) the reaction condition is mild, reacts at room temperature, and requires no extra energy consumption (even no stirring); (c) the use of organic solvents, hazardous agents, or surfactants is avoided; (d) the synthesis procedure is very simple, just by mixing two aqueous solutions; (e) the luminescences are strong and tunable by changing the concentrations of metal salt solution; and (f) the resulting products can exist in the form of liquid and solid states, which are flexible to create complex patterns. In addition, these as-prepared clusters were also used to detect H_2_O_2_, which shows a high sensitivity with a limited detection of 1.0 × 10^−7^ M.

## Results and discussion

**Figure 1:**
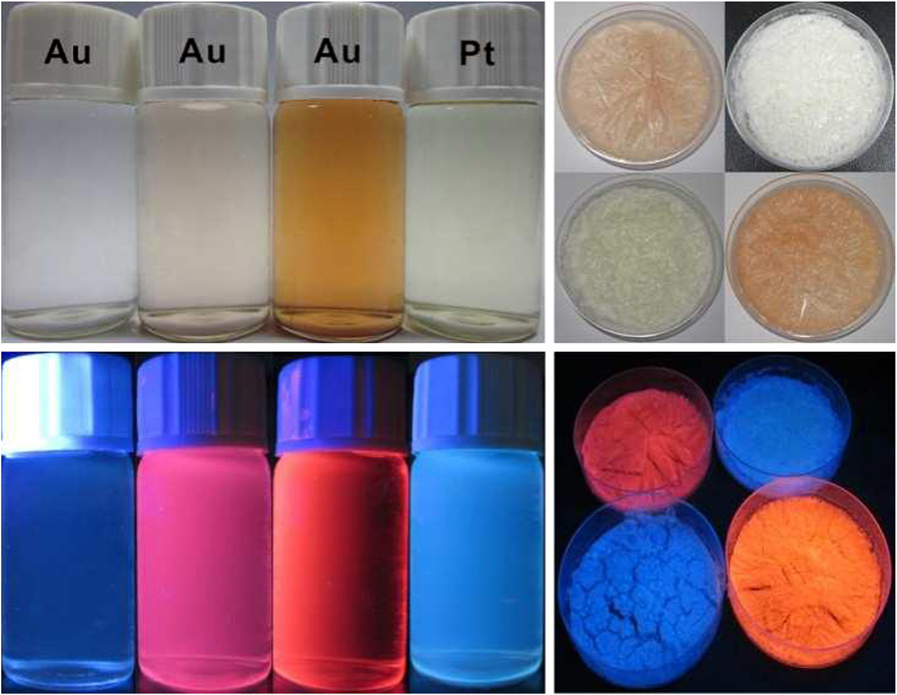
**Photographs of luminescent metal clusters in the form of solution and solid states.** Under natural light and UV irradiation (*λ* = 365 nm).

The photographs of as-prepared products (left: solution form, right: solid form) are shown in Figure 1. Under visible light, the solution colors (Au with three increasing concentrations and Pt) are colorless, pale yellow, deep brown, and pale green, respectively, as shown at the upper left of Figure 1. Correspondingly, under UV light (365 nm), the intense luminescences from them (under a 365-nm UV light) are also observable by naked eyes at the bottom left. Meanwhile, the solid-state luminescences are shown in the right part. Clearly, both forms indicate strong luminescence under UV light.

**Figure 2:**
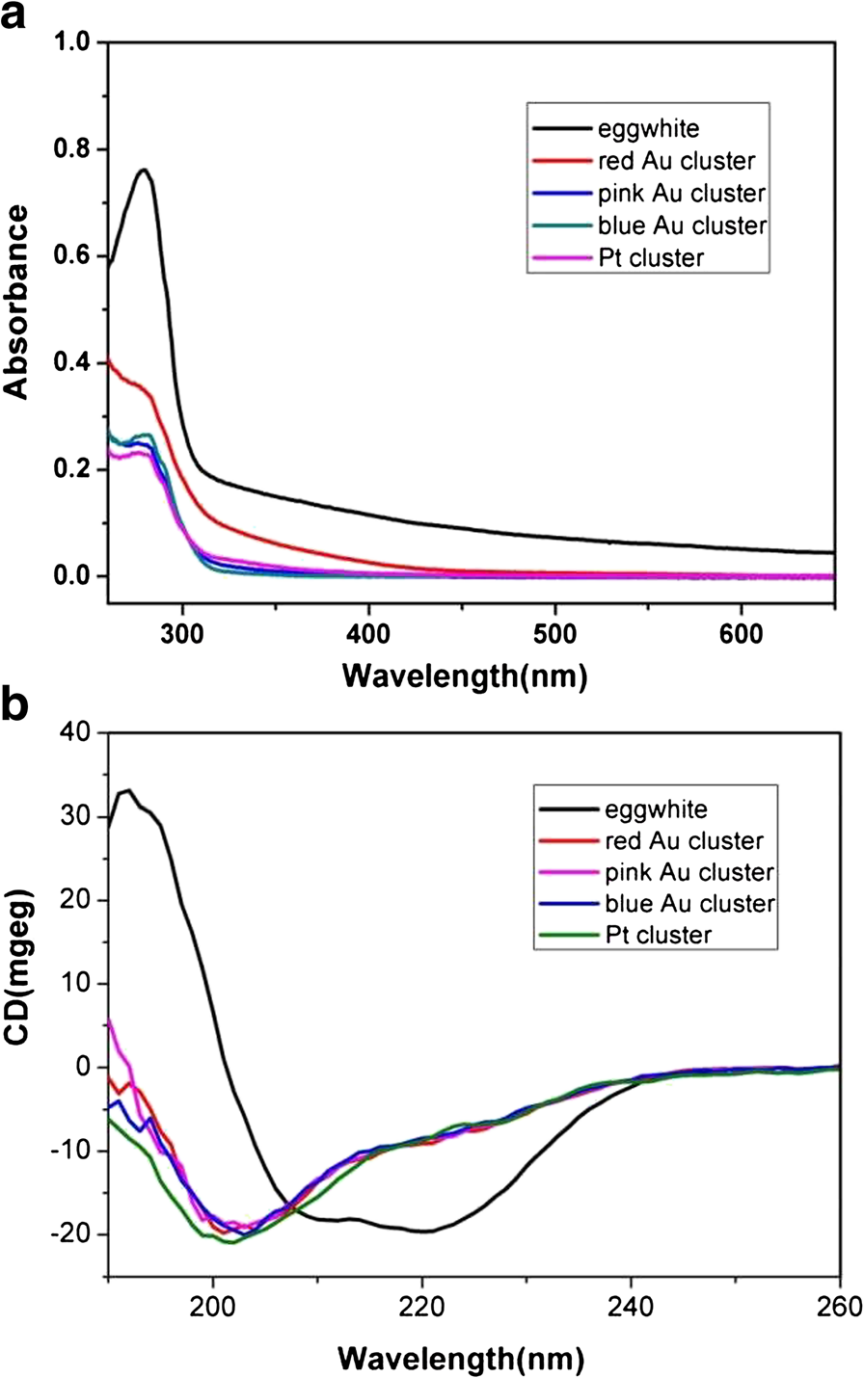
**Spectral Analysis of aqueous solution of chicken egg white and metal clusters.** (**a**) UV-vis absorption spectra; (**b**) CD spectra.

As well known, metal clusters show obviously different absorption features compared to their corresponding nanoparticles. As shown in Figure 2a, the UV absorption spectra of these sample solutions prepared at various Au^3+^ concentrations did not indicate any formation of AuNPs due to the absence of localized surface plasmon resonance bands (*ca.* 520 nm). The absorption peaks at 280 nm could be attributed to the features of aromatic amino acids in proteins. Due to the addition of exogenous agents, the absorption profile of Au and Pt at 280 nm is relatively wider than that of pure egg white, indicating that the variation of the microenvironment has an evident effect to protein conformations. Since circular dichroism (CD) is a kind of effect tool to study proteins’ conformational changes, therefore, we performed CD spectroscopy to reveal their secondary structure changes in detail before and after the formation of metal clusters. As shown in Figure 2b, the CD spectrum of pure egg white aqueous solution displays a negative band around 215 nm and a positive band around 195 nm from the β-sheet as the main structures. However, a negative band around 200 nm from the random coil structure was dominantly observed for the egg white-templated metal clusters. The conformational change indicates that egg white has given rise to denaturation due to the addition of metal ions and strong base.

**Figure 3:**
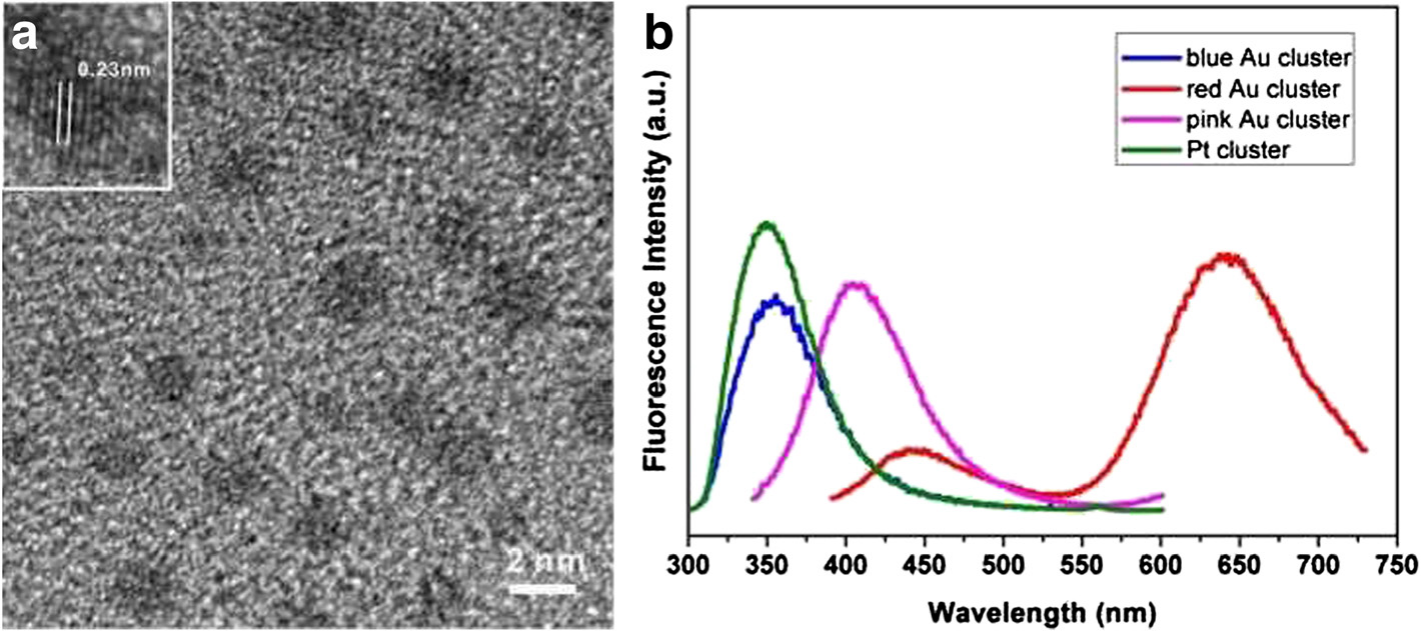
**HRTEM imaging of red-luminescent Au clusters and emission spectra of Au and Pt clusters.** (**a**) HRTEM imaging of red-luminescent Au clusters. (**b**) Emission spectra of red-luminescent, pink-luminescent, and blue-luminescent Au clusters and blue-luminescent Pt cluster.

The high-resolution transmission electron microscope (HRTEM) image showed the presence of metal clusters in the size of approximately 2.5 nm (in diameter) for red-emitting Au (Figure 3a), where the crystal lattice fringes are 0.23 nm, which correspond to the (111) planes of the metallic Au. We deduced that the larger sizes could be due to the continuous irradiation of high-energy electron beams, which leads to the aggregation of the clusters. We failed to observe these dark spots in the HRTEM images of pink-emitting Au, blue-emitting Au, and blue-emitting Pt, which could be attributed to their ultra-small sizes. The fluorescence emissions of the four samples are also shown in Figure 3b. A broad emission maximum at approximately 650 nm for red-luminescent Au (red curve) was shown when the 380-nm exciting wavelength is used. The broad emission could be attributed to the multiple cluster size distributions or the intricate chemical environments around the metal core as pointed out by Xavier et al. [18]. Additionally, a front emission peak at approximately 450 nm was also observed, which is confirmed to be from the egg white (data not shown). The pink-luminescent Au (pink curve) shows an emission maximum at approximately 410 nm (excitation wavelength 330 nm). The blue-luminescent Au (blue curve) and blue-luminescent Pt (green curve) show nearly the same emission maximum at approximately 350 nm. In fact, we also attempted to synthesize Ag clusters using an identical method; after 1 h of incubation, the red luminescence reached the maximum, and afterward, it gradually faded until no luminescence was observed (Additional file 1: Figure S1).

Regarding the formation mechanism, as put forward by Xie et al. [19], with the egg white as stabilizing host material providing a confined space that limits cluster growth and impedes agglomeration, the formation process consists of the trapping and interacting of metal ions, followed by reduction and growth at highly alkaline pH. During the process, the aromatic amino acids in proteins would donate electrons to reduce metal ions; meanwhile, the broken disulphide bonds would stabilize these nucleated clusters. Considering the complexity of proteins in egg white, it might take us a long time to make the mechanism clear. In spite of this, some questions remain haunting us, such as the following: What happened during the 12 h of evolution of clusters in the mixed proteins [28]? Is one or more proteins involved in the formation of metal clusters? What is the number of metal atoms in the cluster core? Is it possible to synthesize metal clusters using plant or animal extracts by adopting a similar method [29-31]? What is the luminescent mechanism of metal clusters in mixed proteins? Further work in our group is being actively explored towards these questions.

There are many reports about fabricating luminescent sensors based on metal clusters [32-35]. Herein, the as-prepared Au clusters were also used as a highly sensitive sensor for the identification of H_2_O_2_, which is a kind of important small-molecule compounds in the environment and bioanalytical sciences. We found that the luminescence of the Au cluster is quenched in the presence of H_2_O_2_.

**Figure 4:**
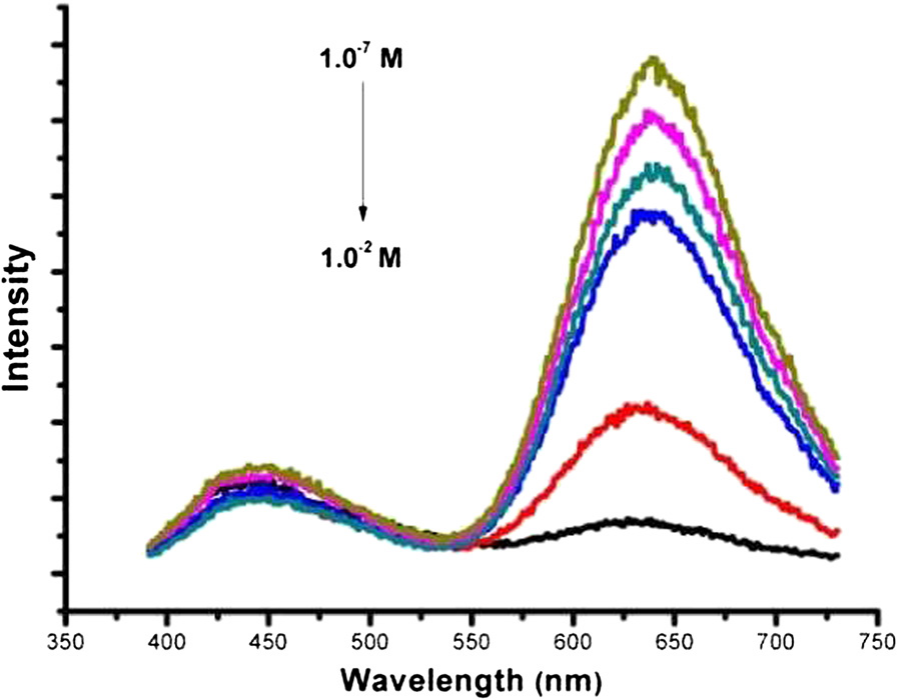
**Fluorescence quenching of red-luminescent Au clusters by the addition of different concentrations of H**_**2**_**O**_**2**_**.** (black) 1.0 × 10^−2^ M, (red) 1.0 × 10^−3^ M, (blue) 1.0 × 10^−4^ M, (green) 1.0 × 10^−5^ M, (pink) 1.0 × 10^−6^ M, (yellow) 1.0 × 10^−7^ M.

From Figure 4, one can see that more and more quenching occurs with increasing H_2_O_2_ concentrations. The quenching mechanism could be attributed to the strong oxidative ability of H_2_O_2_, which disrupted the egg white-protected Au clusters, leading to their aggregation and growth, becoming larger Au nanoparticles. The destructive products were also imaged by TEM (Additional file 1: Figure S2).

## Conclusions

In conclusion, we have developed ‘a real green way’ to synthesize noble metal clusters (Au and Pt) by using chicken egg white as template. The method is simple; source-, energy-, and cost-effective; and environmentally friendly. The resulting products show high luminescence and stability both in the form of liquid and solid states. The red-emitting Au clusters show high sensitivity to H_2_O_2_. By using our method, the commercial production in scale is feasible. We believe that the egg white-templated noble metal clusters (Au and Pt) will find important application potentials in the field of catalysis, bioimaging (contrast agents), biolabeling, sensors, and optoelectronic devices. Following this, some interesting ideas are also suggested: since egg white can also be used for the preparation of vaccines, it seems that our method could throw insight into the development of multi-functional vaccines as well as some multi-functional food additives and antibacterial agents. We will expect a bright future for these clusters.

## Competing interests

The authors declare that they have no competing interests.

## Authors’ contributions

ML and DXC conceived and designed the experiments. ML and DPY performed the experiments. ML, DPY, and XSW analyzed the data. JXL and DXC contributed the materials and analysis tools. LM and DPY wrote the manuscript. All authors read and approved the final manuscript.

## Supplementary Material

Additional file 1 **Experimental.** The file contains the ‘Experimental’ section which discusses the materials and reagents, preparation of Au clusters, and characterization, with Figures S1 and S2. Click here for file
