# Supplementary material for: Mixed protein-templated luminescent metal clusters (Au and Pt) for H2O2 sensing
Source: Nanoscale Res Lett. 2013 Apr 19;8(1):182. doi: 10.1186/1556-276X-8-182 (PMC3643886; doi:10.1186/1556-276X-8-182)
Supplement: Additional file 1 — Experimental. The file contains the ‘Experimental’ section which discusses the materials and reagents, preparation of Au clusters, and characterization, with Figures S1 and S2. [file 1556-276X-8-182-S1.rtf]

Mixed Proteins-Templated Luminescent Metal Clusters (Au and Pt) For H2O2 Sensing

Min Li a, b, Da-Peng Yang* b, Xiansong Wang b , Jianxin Lu* a and Daxiang Cui* b

a Key Laboratory of Laboratory Medicine, Ministry of Education, School of Life Science, Wenzhou Medical School, Wenzhou, China. Fax: 86 0577 86689771; Tel: 86 0577 86689771;

bDepartment of Bio-Nano-Science and Engineering, Shanghai Jiaotong University, Shanghai, China. Fax: 86 021 34206886; Tel: 86 21 34206375; E-mail: dxcui@sjtu.edu.cn; 

Experimental

Materials and Reagents: The fresh chicken eggs were purchased from local market and were broken in a glass dish. The egg-whites were carefully isolated from the mother solution and stored at 4�Ž for the following use. Sodium hydroxide (NaOH), Hydrogen tetrachloroaurate(III) (HAuCl43H2O), Hydrogen hexachloroplatinate(IV) (H2PtCl6), and Silver nitrate (AgNO3) were purchased from Sinopharm Company (China). Millipore water was used throughout all the experiments.

Preparation of Red-luminescent Au clusters

In a typical experiment, 5.0 mL of 10 mM HAuCl4 solution was added to 5.0 mL of fresh chicken

egg-white solution (diluted using H2O, 1:1) at room temperature. The solution became turbidity

immediately. It was then mixed for 2 min. Subsequently, 1.0 ml of 1.0 M NaOH was added into the reaction mixture and the color became colorless immediately. Then, the resulting solution was left undisturbed at room temperature for 24 h incubation and became reddish brown. The solid powder was prepared through subjected to freeze-drying.

Preparation of Pink-luminescent Au cluster

The method is analogous with the preparation of red-luminescent Au cluster. Typically, 5.0 mL of 5 mM HAuCl4 solution was added to 5.0 mL of fresh chicken egg-white solution (diluted usingH2O, 1:1) at room temperature. The solution became turbidity immediately. It was then mixed for 2 min. After 1.0 ml of NaOH (1 M) was added into the above aqueous solution, the color turned into colorless at once. The solution was then allowed to place at room temperature without any disturbance. After 24 h incubation, the color of the resulting solution became pale yellow. The

ChemComm	Page 8 of 9


solid powder was prepared through subjected to freeze-drying.

Preparation of Blue-luminescent Au cluster:

The experimental procedure is as same as the preparation of red-Au and blue-Au clusters except the concentration of HAuCl4 (1.0 mM).

Preparation of Blue-Luminescent Pt Cluster

In a typical experiment, aqueous HPtCl6 solution (5 mL 1.0 mM) was added to fresh chicken egg white solution (5 mL; original eggwhite diluted using water 1:2). The solution was put at room temperature for 2 min, and then 1.0 mL NaOH (1 M) solution was introduced. The color of the solution changed from yellow to colorless. Finally, the mixture was heated in a water bath for 12 h

at 50�Ž, and the resulting color was a little pale yellow. The solid powder was prepared through subjected to freeze-drying.

Characterization

The UV-visible spectra were recorded in a Shimadzu UV-2450 spectrophotometer. Fluorescence measurements were performed on a Hitachi F-4600 fluorescence spectrophotometer (Tokyo, Japan). The microstructure of the clusters was characterized by a JEOL JEM-2010 transmission electron microscope operating at an accelerating voltage of 200 kv. TEM samples were prepared by spraying a dispersion of Au clusters onto a Cu gird covered by a holey carbon film.

Fig. S1. Photographs of egg white-protected Ag cluster under natural light and UV irradiation (365 nm).


Fig. S2. TEM image of H2O2 oxidized Au clusters, showing the occurrence of Au nanoparticles

due to the aggregation and fusion.
